# Metalloproteomic Investigation of Hg-Binding Proteins in Renal Tissue of Rats Exposed to Mercury Chloride

**DOI:** 10.3390/ijms25010164

**Published:** 2023-12-21

**Authors:** Emerson Carlos de Almeida, Victor Diego Faria, Felipe Dalmazzo Cirinêu, Maria G. A. Santiago, Beatriz Miotto, José C. S. Vieira, Camila Pereira Braga, Jiri Adamec, Ana A. H. Fernandes, Marília A. R. Buzalaf, Pedro de Magalhães Padilha

**Affiliations:** 1 Institute of Biosciences, São Paulo State University (UNESP), Botucatu 18618-687, SP, Brazil; emerson.carlos@unesp.br (E.C.d.A.); v.faria@unesp.br (V.D.F.); felipe.cirineu@unesp.br (F.D.C.); m.santiago@unesp.br (M.G.A.S.); beatriz.miotto@unesp.br (B.M.); cavalcante.vieira@unesp.br (J.C.S.V.); ana.ah.fernandes@unesp.br (A.A.H.F.); 2 Department of Redox Biology, University of Nebraska, Lincoln, NE 68198, USA; braga_cap@yahoo.com; 3 School of Medicine, Louisiana State University Health Sciences Center (LSUHSC), New Orleans, LA 70112, USA; jadame@lsuhsc.edu; 4 Faculty of Dentistry of Bauru (FOB), University of Sao Paulo (USP), Bauru 17012-901, SP, Brazil; mbuzalaf@fob.usp.br

**Keywords:** mercurial species, mercury bioaccumulation, mercury biomarkers, metallomics, LC-MS/MS

## Abstract

Results obtained from rat studies indicate that, even at low concentrations, mercurial species cause harmful effects on the kidneys, by inducing the nephrotic oxidative stress response. In the present work, Hg-associated proteins were identified as possible mercury-exposure biomarkers in rat kidneys exposed to low mercury chloride concentrations for 30 days (Hg-30) and 60 days (Hg-60), using metalloproteomic strategies. The renal proteomic profile was fractioned by two-dimensional electrophoresis and the mercury determinations in kidney samples, protein pellets and protein spots were performed using graphite furnace atomic absorption spectrometry. The characterization of Hg-associated protein spots and the analysis of differentially expressed proteins were performed by liquid chromatography, coupled with tandem mass spectrometry. Eleven Hg-associated protein spots with a concentration range of 79 ± 1 to 750 ± 9 mg kg^−1^ in the Hg-60 group were identified. The characterization and expression analyses allowed the identification of 53 proteins that were expressed only in the Hg-60 group, 13 “upregulated” proteins (*p* > 0.95) and 47 “downregulated” proteins (*p* < 0.05). Actin isoforms and hemoglobin subunits were identified in protein spots of the Hg-60 group, with mercury concentrations in the range of 138 to 750 mg kg^−1^, which qualifies these proteins as potential mercury-exposure biomarkers.

## 1. Introduction

Toxic metals (e.g., Cd, Pb, Cd and Hg) are present in various forms in the environment. Humans and animals can be intoxicated by these xenobiotics, mainly through the consumption of contaminated food and water [1]. In the mercury case, various molecular/ionic forms cause deleterious effects to human and animal health through various mechanisms. Among the deleterious effects of mercurial species, the following can be mentioned: interruption of microtubule formation, altering the balance of intracellular calcium and membrane potential, altering the integrity of the cell membrane, disrupting or inhibiting enzymes, inducing oxidative stress, inhibiting protein and DNA synthesis, and disrupting immune functions [2]. When absorbed by the body, mercurial species bind mainly to sulfhydryl groups, but also to phosphoryl, carboxyl and amide groups in biological molecules [1,2,3]. A proteomic study involving one-dimensional electrophoresis of human erythrocytes exposed to concentrations in the range of 1–60 µmol L^−1^ of mercuric chloride, indicated that exposure to Hg^2+^ altered the electrophoretic profile of cytosolic proteins, with a significant decrease in the intensity of the hemoglobin monomer; this was associated with the appearance of a 64 kDa band, identified as a tetrameric form associated with mercury [4]. This finding allows us to infer that Hg^2+^ can cause structural and metabolic changes in erythrocytes, which can cause significant changes in the physiology and morphology of these cells [4]. A subsequent study performed by the same researcher group [4,5] demonstrated the effectiveness of phenolic compounds from olive oil in preventing metabolic and morphological damage in human red blood cells exposed in vitro to treatment with HgCl_2_. The results of the study reinforce the fact that the properties of bioactive compounds in olive oil can prevent the harmful effects of human exposure to mercurial species [5]. Organic mercurial species (methyl/dimethylmercury) are more toxic to the body than the inorganic species (Hg^2+^ or Hg^0^), and may induce oxidative stress and consequently generate free radicals, causing neurotoxicity. There are also reports that the accumulation of serotonin, aspartate, and glutamate possibly plays a role in the mechanism of methyl mercury induced-neurotoxicity [2,6]. 

In relation to the central nervous system (CNS), a probable mechanism of action of mercurial species is the conversion of methylmercury to an inorganic form, which subsequently binds to thiol groups of proteins. Both organic and inorganic forms of mercury are soft acids, and thus show high affinity for thiol groups (soft bases) of proteins, such as cysteine [7,8,9]. Low-level exposure for long periods to mercury salts (i.e., HgCl_2_) and/or mercury organic species (e.g., methylmercury) cause acute symptoms in the gastrointestinal tract (GIT), late neurotoxicity and nerve cell destruction [2,3,8]. 

Color change in the mucous membranes, oropharyngeal pain, nausea, vomiting, bloody diarrhea, abdominal colic and renal dysfunction are the main symptoms of intoxication by inorganic mercury compounds [2,10]. When absorbed into the bloodstream, approximately 90% of inorganic mercury species accumulate in the renal tissue; in this case, they are absorbed, and accumulate in the proximal tubules of the kidneys [2,11,12]. The main symptoms of intoxication are polyuria and proteinuria, which indicate tubular damage in the kidneys. In the case of severe intoxication, nephrotic syndrome and anuria may occur, and in chronic intoxications, membranous nephropathy may occur [2,12].

The kidneys, as the main route of excretion of organisms, are more susceptible to toxic xenobiotics, mainly including metals and/or metalloids, such as Pb, Hg, Cd and As. In addition, depending on the dose and time of exposure, toxic metals can potentiate toxicity at intracellular sites in the liver and kidney [1]. A study of mercuric chloride exposure in rats demonstrated that there is no uniform organ distribution after absorption of Hg^2+^ ions, and that these ions accumulate mainly in the kidneys, causing acute renal failure. In this case, Hg^2+^ ions accumulate in renal proximal tubular cells and induce cell injury by binding and inhibiting enzymes containing sulfhydryl groups [13,14]. Access to tubular cells by Hg^2+^ ions is mainly available through amino acid transporters in the luminal plasma membrane and by organic anion transporters (Oat1 and Oat3) in the basolateral plasma membrane [1,13]. Due to the binding to the sulfhydryl groups of Hg^2+^ ions, the oxidation of these groups and inactivation of the enzyme may occur, resulting in the depletion of the total content of sulfhydryl groups and oxidative stress [1,14,15]. Inactivation of enzymes with sulfhydryl groups affects cell integrity, and consequently disrupts the membrane potential and the cell and organelle volume [1,15,16,17]. 

In general, studies of the absorption and toxicity of mercurial species by human and animal organisms are mostly centered on monitoring mercury concentrations and the correlation with histochemical and gene expression studies. There are still few studies that report on using proteomics tools, which may allow post-translational information related to the toxicity of mercurial species. In this context, metalloproteomics, a line of research that consists of the integration of analytical and biochemical studies, can contribute important information regarding the interaction of metallic species with macromolecules. The physiological and functional aspects of metalloproteins and/or metal binding proteins responsible for numerous biological processes and molecular functions have been studied with metalloproteomic approaches in recent years [9,18,19,20]. Toxicity studies of mercurial species report that metalloproteomics allows the identification of proteins differentially expressed in protein spots of biological samples (e.g., liver and muscle tissues), in view of human and animal exposure to mercurial species, providing information regarding the mechanisms of mercury absorption by living organisms [19,20].

Based on the above, this paper presents metalloproteomic data from the rat kidneys, which were obtained after the exposure of rats to low concentrations of mercury chloride, and which allowed the characterization of Hg-binding proteins as a possible mercury-exposure biomarker (i.e., Hg^2+^).

## 2. Results and Discussion

### 2.1. Hg-Determinations

The Hg-determination results in Table 1 show that an increase in the exposure time of rats to mercury chloride contributed to the process of mercury bioaccumulation in the renal tissue. In relation to the C-60 group, GFAAS determinations did not detect the presence of mercury. The results presented in the table show an increase of approximately 54% in the Hg concentration in the Hg-60 group (60.40 ± 1.10 µg kg^−1^), compared to the Hg-30 group (39.20 ± 0.71 µg kg^−1^). The same behavior can be observed in relation to the results of the protein pellets; in this case, the Hg concentration in the pellets of the Hg-60 group (55.60 ± 0.98 µg kg^−1^) was approximately 52% higher than that of the Hg-30 group (36.50 ± 0.62 µg kg^−1^). The analysis of DOLT 4-certified material presented a recovery percentage of 98% (Table 1), thus validating the total Hg determinations in the samples. Considering that the Hg concentration injected into the rats in the Hg-30 treatment was 6.07 µg kg^−1^, and that the Hg concentration determined in the kidney was 39.20 ± 0.71 µg kg^−1^ (Table 1), the Hg bioaccumulation in the rat kidneys was approximately 646%. Regarding the Hg-60 group, the Hg concentration injected into the rats was 8.02 µg kg^−1^, and the Hg concentration determined in the kidney was 60.40 ± 0.80 µg kg^−1^. Under these conditions, the Hg bioaccumulation in the rat kidney was 753%. Thus, the effect of exposure frequency directly affects the Hg bioaccumulation in rat kidney.

### 2.2. Renal Proteome Fractionation

**Figure 1:**
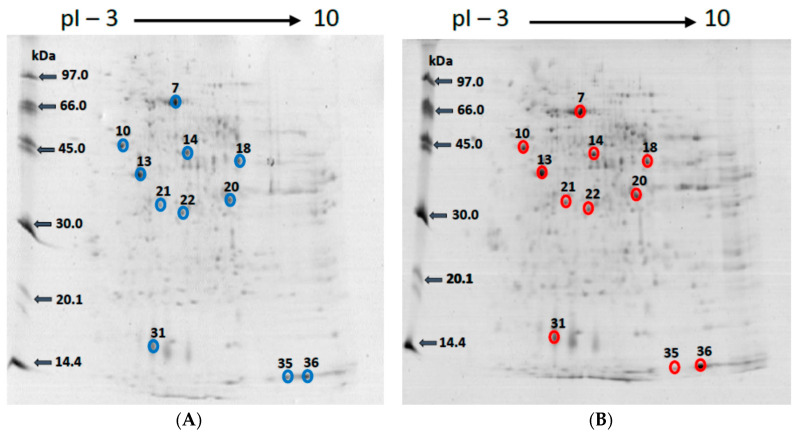
Representative results of triplicates analyses of renal tissues samples by 2D-PAGE from rats in the control groups ((**A**), C-60) and those exposed to mercury for a period of 60 days ((**B**), Hg-60). Spots marked with blue circles (**A**) are those in which GF-AAS determinations have not indicated the presence of mercury. In the spots marked with red circles (**B**), the GFAAS determinations indicated the presence of mercury.

Considering that the mercury bioaccumulation process was more effective in the Hg-60 group, the renal proteome of the C-60 and Hg-60 groups was fractionated by 2D PAGE, with the objective of identifying mercury-associated protein spots through determinations by GFAAS. As an example, Figure 1 shows one 2D PAGE gel from each replicate of the C-60 and Hg-60 groups. The triplicate analyses of 2D PAGE gels indicated that each gel presents 190 ± 8 spots—Matching = 95 ± 5% (C-60 group) and 253 ± 8 spots—Matching = 99 ± 1% (Hg-60 group). The GFAAS analyses performed to map the presence of mercury in protein spots indicated concentrations in the range of 79 ± 1 to 750 ± 9 mg kg^−1^ in 11 spots (highlighted in red in Figure 1). The results are summarized in Table 2.

### 2.3. Characterization of Hg-Associated Protein Spots by LC–MS/MS

The characterization analyses of 11 Hg-associated protein spots by LC–MS/MS identified 12 proteins in spots 7, 13, 22, 31, 35 and 36 (Hg-60 group). The proteins and/or enzymes identified are shown in Table 2.

In spot 7, albumin was identified, a protein whose main function is to regulate the colloidal osmotic pressure of the blood [21]. Spot 7 presented a 120 mg kg^−1^ mercury concentration (Table 2). A paper by Li et al. [22], using online capillary electrophoresis coupled with electrothermal-atomization atomic absorption spectrometry (EC-ETAAS) and structural information from circular dichroism and Raman spectroscopy confirmed the binding of mercurial species (Hg^2+^, MeHg^+^) with human serum albumin (HSA). Considering the high Hg concentration in spot 7, it can be inferred that the albumin identified in this protein spot of the renal proteome of rats (Hg-60 group) has the potential characteristic of an exposure biomarker for mercury species.

Beta 1 and 2 isoforms of hemoglobin were identified in spots 13, 35 and 36, spots that presented 138 ± 2 and 84 ± 1 mg kg^−1^ of Hg, respectively. The beta 1 and 2 subunits of hemoglobin are responsible for transporting oxygen from the lung to the various peripheral tissues [21]. The literature reports that, due to the cysteine residue found in hemoglobin isoforms, mercurial species can form coordinative bonds with this protein [20]. A study by Gibson et al. 2017 [23], using a size-exclusion chromatography coupled to inductively coupled plasma atomic emission spectrometry (SEC-ICP-AES) system investigated probable biochemical mechanisms of Cd^2+^, Hg^2+^, CH_3_Hg^+^ ions in rabbit red-blood-cell lysates. The results of this study revealed that the interaction of Hg^2+^ with hemoglobin (Hb) resulted in the formation of a comparatively stable (Hb) XHg complex. Considering the high Hg concentrations determined in spots 13 and 36, in which hemoglobin beta 1 and 2 subunits were identified, it could be inferred that these hemoglobin isoforms act as mercury-exposure biomarkers.

In spot 22, determinations by GFAAS indicated a concentration of 750 ± 9 mg kg^−1^ of Hg, which was the highest Hg concentration determined in protein spots. In spot 22, six isoforms of actin were identified: cytoplasmic actin 1 and 2, alpha skeletal muscle actin, alpha 1 cardiac muscle actin, gamma-enteric smooth muscle actin and aortic smooth muscle actin. Actins are involved in various types of cell motility, and are ubiquitously expressed in all eukaryotic cells [21]. Recently, a study by Nong et al., 2021 [24], using chromatographic techniques (SEC) coupled with atomic spectrometry (ICP–MS), demonstrated that beta-actin can act as a novel Hg-binding protein in fish muscle tissue, and that 30–37% of Hg present in fish muscle tissue is bound to beta-actin. The high Hg concentration determined in spot 22, and the results of the recent work by Nong et al. 2021 [24], corroborate the inference that actin and its isoforms present great potential as exposure biomarkers for mercurial species.

Major urinary proteins (Mups) were identified in spot 31, a spot that has 349 ± 5 mg kg^−1^ of total mercury. Mups bind and release pheromones, protect pheromones from oxidation, and may act to regulate social behaviors such as aggression, mating, nursing, territory establishment, and dominance [21]. Mups have a structure with eight beta sheets arranged in an antiparallel beta barrel open on one side, with helices alpha at both ends, and have terminal methionine in their peptide sequences [21,25]. No evidence has been reported which indicates any binding of mercurial species to the Mup structure. However, the Mups have cysteine and methionine in their peptide sequences, amino acids that present basic sites that are very reactive to mercurial species. Cysteine presents free sulfhydryl groups (soft base), while methionine presents thioether groups with two non-bonding pairs of electrons (soft base, too). The sulfhydryl groups can form covalent bonds with soft acids, and thioether groups can form coordinative bonds with soft bases, which may explain the high Hg concentration (soft acid) determined in spot 31 [7]. Based on the results of the present study, it can be inferred that Mups present characteristics of an exposure biomarker for mercurial species.

### 2.4. Analysis Using the Shotgun-LC–MS/MS Strategy

**Figure 2:**
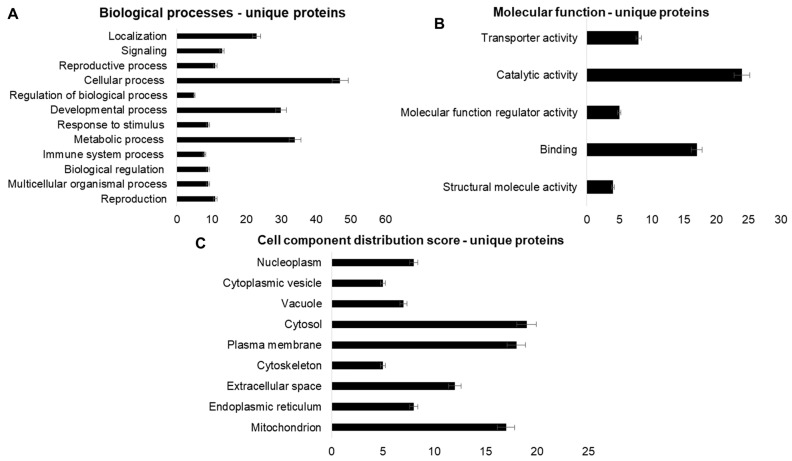
Functional analysis graphs of proteins identified as “unique” in renal tissue samples from rats exposed to mercury for a period of 60 days (Hg-60 group), using Blast2GO 6.0 software. Graph (**A**)—Biological processes; Graph (**B**)—Molecular function; Graph (**C**)—Cell components.

**Figure 3:**
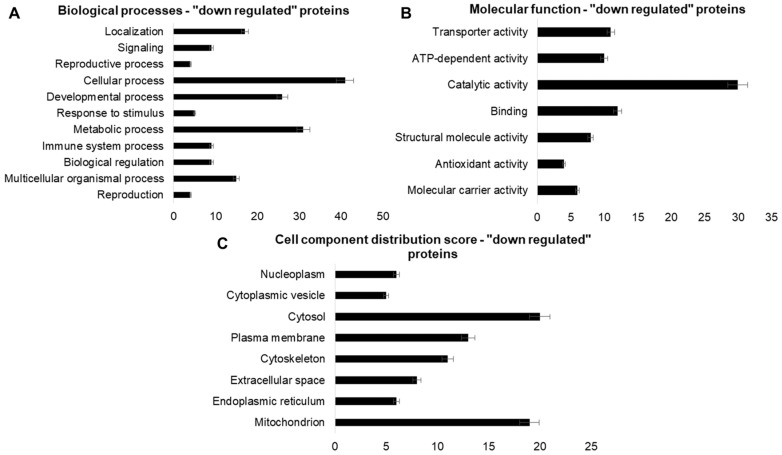
Functional analysis graphs of proteins identified as “downregulated” in renal tissue samples from rats exposed to mercury for a period of 60 days (Hg-60 group), using Blast2GO 6.0 software. Graph (**A**)—Biological processes; Graph (**B**)—Molecular function; Graph (**C**)—Cell components.

**Figure 4:**
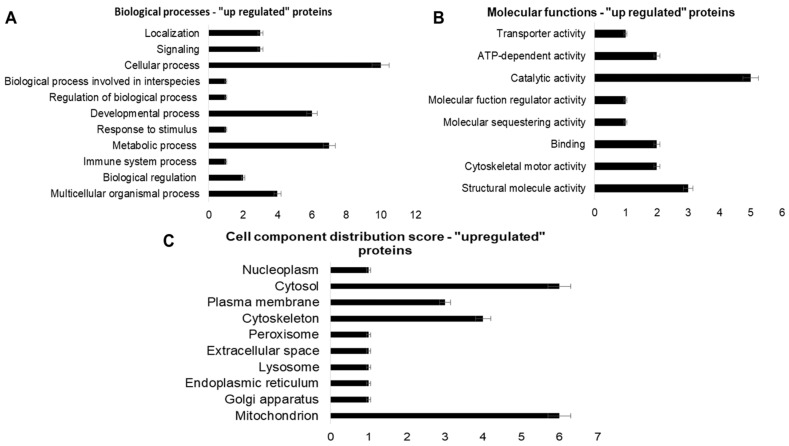
Functional analysis graphs of proteins identified as “upregulated” in renal tissue samples from rats exposed to mercury for a period of 60 days (Hg-60 group), using Blast2GO 6.0 software. Graph (**A**)—Biological processes; Graph (**B**)—Molecular function; Graph (**C**)—Cell components.

The differentially expressed protein analysis was performed using the shotgun–LC–MS/MS strategy, by comparing the C-60 group with the Hg-60 group (C-60 × Hg 60). The results obtained are summarized in Tables S1–S3 (Supplementary Materials). Blast2GO 6.0 software was used in the functional analysis of proteins that showed significant “fold change” or that expressed themselves in a single group (C-60 or Hg-60). The results obtained are shown in Figure 2, Figure 3 and Figure 4. The expression analysis by shotgun—LC–MS/MS identified 52 unique proteins (30 proteins “expressed only in the control group and 20 in the Hg-60 group”, 13 “upregulated” proteins (*p* > 0.95) and 47 “downregulated” proteins (*p* < 0.05). Regarding the functional analysis (Figure 2, Figure 3 and Figure 4) of the proteins and/or enzymes identified as unique, with “upregulated” and “downregulated” (C-60 group × Hg-60 group), the results indicate that these proteins/enzymes participate in important biological processes, as well as cellular components and molecular functions.

Briefly, considering the proteins that were “expressed only in the C-60 and Hg-60 group” (Figure 2, Figure 3 and Figure 4), it can be observed that, in relation to biological processes, these proteins present 11 peptide sequences involved with reproduction processes; 9 with multicellular organism processes; 9 with biological regulation processes; 8 with immune system processes; 34 with metabolic processes; 9 with stimulus–response processes; 30 with development processes; 13 with signaling processes and 23 with localization processes. Regarding molecular function, they presented 4 peptide sequences involved in structural molecular activity, 17 involved in binding, 5 involved in regulatory activity of molecular function, 24 involved in catalytic activity and 8 involved in transport activity. In relation to the cellular component, they present 51 FASTA sequences of anatomical entity, distributed as follows: 17 from mitochondria; 8 from endoplasmic reticulum; 12 from extracellular space; 5 from cytoskeleton; 18 from plasmatic membrane; 19 from cytosol; 7 from vacuole; 5 from cytoplasmic vesicle and 8 from nucleoplasm.

In relation to the “downregulated” proteins, these present the following peptide sequences: biological processes: 4 of reproduction; 15 of process of multicellular organism; 9 of biological regulation; 9 of immune system process; 31 of metabolic process; 5 stimulus– response; 26 of development process; 41 of cellular processes; 4 of reproductive processes; 9 for signaling and 17 for location. Molecular function: 6 molecular carrier activity sequences; 4 antioxidant activity; 8 structural molecular activity; 12 binding; 30 catalytic activity; 10 ATP-dependent activity and 11 transport activity. Cellular component: 100% of the peptide sequences (43) perform functions related to cellular anatomy, distributed as follows: 19 from mitochondria; 6 from endoplasmic reticulum; 8 extracellular space; 11 of cytoskeleton; 13 of plasma membrane; 20 µg cytosol; 5 from cytoplasmic vesicle and 6 from nucleoplasm.

Regarding the “upregulated” proteins, they present the following peptide sequences. Biological processes: four process sequences from multicellular organisms; two of biological regulation; one of immune-system process; seven of metabolic process; one stimulus–response; six of developmental process; one biological process involved in the interaction of interspecific organisms; ten of cellular process; three for signaling and three for location. Molecular function: three sequences of structural molecular activity; two of cytoskeletal-motor activity; two binding; one for molecular-scavenging activity; one of molecular-function regulatory activity; five for catalytic activity; two of ATP-dependent activity and one of transport activity. Cellular component: the 13 “upregulated” sequences are part of a cell structure, distributed as follows: six from mitochondria; one Golgi complex, one endoplasmic reticulum; one lysosome; one extracellular space; one peroxisome; four of cytoskeleton; three of plasma membrane; six from cytosol and one from nucleoplasm.

Based on information from the functional analysis and protein expression, the following proteins/enzymes that were expressed only in the Hg-60 group can be highlighted: isoforms 2, 4 and 7 of glutathione S-transferase Mu (GST Mu), glutathione peroxidase (GPx) and the following heat shock proteins: heat shock 70 kDa protein 1-like and heat shock protein beta-1. A metalloproteomic study by Bitarello et al. 2020 [18] identified the GST Mu and GPx enzymes in the renal and hepatic proteomes of mercury-contaminated Amazonian fish. The authors, through GST and GPx activity analysis, inferred the participation of these enzymes as possible detoxifiers of fish organisms exposed to mercurial species. The selenol groups of GPx act as soft bases, and therefore are quite reactive in relation to soft acids such as Hg^2+^ ions [7,18]. Based on this finding in the literature, it can be inferred that the GST and GPx isoforms were possibly expressed only in the group exposed to HgCl_2_ (Hg-60), due to their detoxifying properties, as a defense of the rat organism against toxic effects of HgCl_2_ [18]. Heat shock 70 kDa protein 1-like and heat shock protein beta-1 belong to the class of “chaperones—HSPs”, proteins that are induced by different types of stressors, such as changes in temperature, salinity, pH, dissolved oxygen and toxic metals such as cadmium, lead and mercury, which may explain the fact that HSPs were only expressed in the group exposed to HgCl_2_ (Hg-60) [26]. As HSPs participate in defense mechanisms induced under suboptimal physiological conditions, the name “Heat Shock Proteins” was replaced by “Stress Proteins”, as a more general term that better encompasses their range of action [26].

Regarding the proteins and/or enzymes that were “upregulated”, actin_alpha from cardiac muscle 1, Calsequestrin-2 and the C region of the Ig gamma-1 chain can be highlighted. Cardiac muscle alpha actin 1 was one of the actin isoforms identified in spot 22 as one of the mercury-associated proteins (as discussed earlier). Spot 22 had a total concentration of mercury equal to 750 mg kg^−1^; thus, considering the significant increase in the expression of actin_alpha cardiac muscle 1 (*p* = 1), we established this protein as a potential biomarker of mercurial-species exposure.

Among the proteins and/or enzymes that were “downregulated”, the following stand out: isoforms 1 and 2 of Actin_ cytoplasmic, Actin_ gamma-enteric smooth muscle, Actin_ aortic smooth muscle and Actin_ alpha skeletal muscle; and beta 1, 2 and alpha ½ isoforms of the hemoglobin subunit. The actin isoforms (according to previous discussion) were identified in spot 22, with a total mercury concentration greater than 700 mg kg^−1^, and the protein expression analyses indicated a “fold change” equal to 0, which allows us to state that actin and its isoforms show characteristics of potential biomarkers of mercury exposure. The beta 1 and 2 isoforms of the hemoglobin subunit also showed “downregulation” equal to 0, and were identified in protein spots 13 and 31, spots with a total mercury concentration equal to 138 and 341 mg kg^−1^, respectively; these data qualify hemoglobin subunits beta 1 and 2 as potential mercury-exposure biomarkers.

## 3. Material and Methods

### 3.1. Animal Experimentation

“Animal experiments were approved by the Ethics Committee on the Use of Animals (CEUA)-UNESP, Botucatu/SP, Brazil, according to protocol number 1179-CEUA. The animals were provided by the Central Animal Facility of UNESP, Botucatu/SP, Brazil, and maintained in the Laboratory of Animal Experimentation of the Institute of Biosciences, UNESP, Botucatu/SP, Brazil [19]. The animal experiments consisted of rearing 36 male Wistar rats (Rattus norvegicus), approximately 40 days old and with an average weight of 250 g, using the procedures described by Santiago et al., 2023 and Rizzetti et al., 2019 [19,27], which are briefly described as follows: the animals were kept in an environment with controlled temperature and photoperiod: 25 ± 2 °C and 12:12 h light/dark cycles, respectively. The feeding was carried out with commercial feed and filtered water, both ad libitum. The animals were divided into four groups: control 30 days (C-30, n = 10), control 60 days (C-60, n = 10), mercury 30 days (Hg-30, n = 10) and mercury 60 days (Hg-60, n = 10). The control groups (30- and 60-days) received an initial dose of 4.60 µg kg^−1^ of 1% saline solution (*m*/*v*) in the first week, and weekly doses of 0.49 µg kg^−1^, following the same procedure used in the mercury chloride solution administration to the mercury-exposed groups, described in and following [27]. The mercury-exposed groups (30 and 60 days) received a dose of 4.60 µg kg^−1^ of mercury chloride (in this case, 4.60 µg kg^−1^ in Hg^2+^) in the first week, and subsequent weekly doses of 0.49 µg kg^−1^ (3 doses of 0.49 µg kg^−1^ in the following weeks for the 30-day group and 7 doses of 0.49 µg kg^−1^ in the following weeks for the 60-day group) [27]. Thus, the total concentration of Hg^2+^ injected into the animals was 6.07 µg kg^−1^ for the Hg-30 group and 8.03 µg kg^−1^ for the Hg-60 group. Saline and mercury chloride solutions were administered intraperitoneally and in the morning (exactly at 6:00 a.m.). The boxes where the rats were kept were cleaned twice a week for 30 or 60 days, and the water and diet were replenished every day”.

### 3.2. Renal Proteome Fractionation by 2D PAGE

At the end of animal experimentation, the animals were weighed, anesthetized and euthanized, according to procedures described by Santiago et al., 2023 [19]. The rat kidneys (whole kidneys) were collected in polypropylene tubes and stored in a freezer at −80 °C. The extraction of protein was carried out using 400 mg of three pools, prepared using kidney tissue from each rat of the control groups (C-30 or C-60) and/or groups exposed to mercury (Hg-30 or Hg-60). The extractions were performed with ultrapure water (18.2 MΩ cm^−1^) in an automated cell disruptor (OMNI BEAD RUPTOR 4, Kennesaw, GA, USA), and using a proportion of 0.40 g tissue to 0.40 mL H_2_O, and with five minutes of homogenization. The suspensions obtained in the extraction process were centrifuged at 10,000 rpm at 4 °C for 15 min, the time required to obtain an extract free of solid concomitants [19]. After obtaining protein extracts, to eliminate possible contaminants present in the extracts, the protein fractions were precipitated using ice-cold acetone (Merck, Rahway, NJ, USA, analytical grade) at 80% (*v*/*v*), in a proportion of 1:4 (extract:acetone), according to the procedure described by Bataglioli et al., 2019 and Santiago et al., 2023 [9,19]. The protein pellets obtained were divided into two parts. One part was used for quantification of total protein, a necessary step to perform calculations of the volume of protein extract containing 375 µg to be applied posteriorly to the IEF strips [9]. The other part of the protein extract was stored in a freezer at −80 °C for the metalloproteomic experiments. The fractionation process of renal proteomes was performed by two-dimensional electrophoresis (2D PAGE). The experimental conditions used in 2D PAGE runs (first dimension—IEF and second dimension—SDS) were described in previously published papers by the research group [18,19,20], in the following way: “For the electrical focusing (IEF) step, which consists of separation by isoelectric point (pI), aliquots of protein extracts were diluted in a solution containing 7 mol L^−1^ urea (Amersham Biosciences, Amersham, UK), 2 mol L^−1^ thiourea (Amersham Biosciences), 2% (mv) CHAPS (Amersham Biosciences), ampholytes (Amersham Biosciences), from pH 3 to 10 at 0.5% (*v*/*v*), bromophenol blue (Amersham Biosciences) at 0.002% (*v*/*v*) and 0.02 mol L^−1^ DTT (J.T. Backer, Phillipsburg, NJ, USA), to adjust the total protein concentrations to 1.50 μg μL^−1^. Three 13 cm IEF strips were hydrated with IPG buffer (with a pH gradient from 3 to 10) and with 250 μL of protein extract containing 1.50 μg μL^−1^ of total protein (which corresponds to 375 μg of protein of each one of the three liver-tissue pools) in their respective channels in the hydration box, and covered with mineral oil to prevent evaporation of the sample solution. The hydration box was closed and kept for 12 h at room temperature. After being properly hydrated, the strips were transferred to the isoelectric focusing system (Ettan IPGphor 3 device, GE Healthcare, Chicago, IL, USA) for the first-dimensional electrophoretic run. The running time was 4.5 h. After the first dimension of the process, characterized by the fractionation of proteins according to their isoelectric point, the IEF strips were rehydrated using two solutions. First, 12 mL of a solution containing 6 mol L^−1^ urea (Amersham Biosciences), 2% (*m*/*v*) SDS (Amersham Biosciences), 30% (*v*/*v*) glycerol (Amersham Biosciences), 50 mmol L^−1^ Tris-HCl (Amersham Biosciences) (pH 8.8), 0.002% (*m*/*v*) bromophenol (Amersham Biosciences) and 2% (*m*/*v*) DTT (J.T. Backer) was added to break the disulfide bonds and keep the proteins in the reduced form. The contact time of the strips with this solution was 15 min on a shaking table. Then, to prevent protein reoxidation and promote alkylation of thiol groups, a solution similar to the previous one, differentiated by the replacement of DTT (J.T. Backer) with 2.5% (*m*/*v*) iodoacetamide (Amersham Biosciences), was added. The contact time with the second solution was also 15 min, on a shaking table. In the second part of the protein fractionation process, based on the separation of proteins by molecular mass using SDS–PAGE, a 12.5% (*m*/*v*) polyacrylamide gel was used. The respective gels were previously prepared from acrylamide (Amersham Biosciences), N,N′-methylenebisacrylamide (Amersham Biosciences), Tris-hydroxymethylamino methane (Amersham Biosciences), sodium dodecyl sulfate (SDS) (Amersham Biosciences), ammonium persulfate (J.T. Backer), N,N′,N,N′tetramethylenediamine (TEMED) (Amersham Biosciences) and ultrapure water. The strips were applied to the surface of the gels, together with filter paper containing 7 μL of protein solution with known molecular mass (Amersham Biosciences): β-phosphorylase (97.0 kDa), albumin (66.0 kDa), ovalbumin (45.0 kDa), carbonic anhydrase (30.0 kDa), trypsin inhibitor (20.1 kDa) and α-lactoalbumin (14.4 kDa). The system was sealed with 0.5% (*m*/*v*) agarose (Amersham Biosciences), to ensure the contact of the gels with the strips in the electrophoretic run. The running time was 4.40 h. The second-dimension runs were performed in Healthcare Life Sciences, using the Ettan™ Dalt Six 2D-PAGE electrophoresis system, Uppsala, Sweden. After the end of the runs, the proteins were fixed in the gels with a solution containing 10% (*v*/*v*) acetic acid (J.T. Backer), and 40% (*v*/*v*) ethanol (J.T. Backer), for 1 h. Then, to visualize the protein spots, colloidal Coomassie dye (J.T. Backer), containing 8% (*m*/*v*) ammonium sulfate (J.T. Backer), 1.6% (*v*/*v*) phosphoric acid (J.T. Backer), 0.08% (*m*/*v*) Coomassie blue G-250 (J.T. Backer), and 25% (*v*/*v*) methanol (J.T. Backer), was added, which remained in contact with the gels for 72 h. To remove the dye, washing with ultrapure water for 30 min for 5 h was necessary. After the completion of the protein-spot-revealing step, the gels were scanned using the ImageMaster 2D Platinum 7.0 program (Uppsala, Sweden), for image processing. The scanned images of the 2D PAGE gels were saved in tif and mel extensions, with a resolution of 300 dpi, zoom 100%, and depth 12 or 16 per pixel, and were then imported into the ImageMaster 2D Platinum 7.0 program (Uppsala, Sweden)”.

### 3.3. Hg Determinations by GFAAS

The Hg determinations in kidney samples, pellets and protein spots were performed using graphite furnace atomic absorption spectrometry (GFAAS), according to the procedures previously described by Moraes et al., 2013 and Bittarello et al., 2020 [18,28]. In the case of protein spots, they were cut out of the respective 2D PAGE gels using a surgical scalpel, and were subsequently transferred to the digestion tubes [18]. The experimental conditions used are briefly described as follows: “the samples (about 100 mg of kidney tissue and 30 mg of protein pellets—weighed in triplicate—and/or three protein spots) were mineralized, using a mixture containing 1.00 mL of concentrated sulfuric acid (18 mol L^−1^, Merck, analytical grade) and 0.50 mL of 30% (*w*/*w*) hydrogen peroxide (Merck, perhydrol, analytical grade) and subjected to gentle heating in a digester block until transparent extracts were obtained. Then, the extracts obtained were made up to 5.00 mL in volumetric flasks with ultrapure water (18.2 MΩ cm^−1^). The determinations of total mercury were carried out by atomic absorption spectrometry in a graphite furnace, using a Shimadzu AA-6800 spectrometer (Kyoto, Japan), equipped with a background absorption corrector with a deuterium lamp and a self-reverse system (SR), a pyrolytic graphite tube with an integrated platform and an ASC-6100 automatic sampler. A Shimadzu hollow cathode mercury lamp was used, and operated at a current of 12 mA. The wavelength was 253.7 nm, and the spectral resolution was 0.5 nm. The absorbance values were measured in peak area. The inner walls of the pyrolytic graphite tubes with integrated platforms used in the mercury analyses were coated with tungsten. For this purpose, 25 μL aliquots of 1000 mg L^−1^ sodium tungstate solution (Merck, analytical grade) were injected into the atomizer, which was then subjected to the program described as follows: Drying—temperature: 95 °C, time = 40 s, 120 °C, time = 5 s, Argon flow (L min^−1^) = 0.30; Reduction—temperature: 500 °C, time = 10 s, 550 °C, time = 5 s, Argon flow (L min^−1^) = 0.30; Cleaning—temperature: 1600 °C, time = 5 s, Argon flow (L min^−1^) = 0.30. Tungsten ions were deposited on the graphite tube platform with heating up to 500 °C, forming a tungsten carbide layer that acted as chemical modifier. We were able to use the graphite tube in 483 firings of the samples after this treatment. The analytical curve was prepared using a stock standard solution of mercury ao 1000 mg L^−1^ (Titrisol^®^ Merck). The mercury standards were prepared using the autosampler. Aliquots of 1.20, 2.40, 4.80, 9.60 and 12.00 μL of a 2.50 μg L^−1^ mercury standard solution (prepared by diluting Titrisol^®^ Merck mercury standard solution) were combined with 4 μL of 100 mg L^−1^ of zirconium chloride solution (Sigma-Aldrich, St. Louis, MO, USA) and sufficient ultrapure water to bring the final volume of the solutions to 20.00 μL. The concentrations of the mercury standard solutions ranged between 0.15 and 1.50 μg L^−1^. These solutions were then injected into the spectrometer’s graphite tube coated with tungsten carbide, using the micropipette of the autosampler. The absorbance measurements were performed in triplicate, and the graphite tube’s heating program, optimized for mercury analysis, is described as follows: Drying—temperature: 95 °C, time = 5 s, 120 °C, time = 5 s, Argon flow (L min^−1^) = 1.00; Pyrolysis—temperature: 250 °C, time = 5 s, 800 °C, time = 5 s, Argon flow (L min^−1^) = 1.00; Atomization—temperature: 1600 °C, time = 5 s, Argon flow (L min^−1^) = 0.00; Cleaning—temperature: 1800 °C, time = 5 s, Argon flow (L min^−1^) = 1.00. Mercury determinations in the samples were made using 20 μL aliquots, obtained by mixing 10.00 μL of acid extract with 4.00 μL of 100 mg L^−1^ of zirconium chloride solution and 6.00 μL of ultrapure water, and were injected into the graphite tube coated with tungsten carbide, using the micropipette autosampler. The measurements were performed in triplicate, using the graphite tube’s heating program described above for standard mercury solutions analysis. All determinations were validated through analysis of National Research Council of Canada (NRC) DOLT 4 certified reference material. In this case, the procedures for analyzing the Dolt 4 certified material were similar to the procedures used for the kidney tissue samples”.

### 3.4. Analysis by LC–MS/MS

#### 3.4.1. Characterization of Hg-Associated Protein Spots

The Hg-associated protein spots were cut from the 2D PAGE gels with a scalpel into segments of approximately 1 mm^3^, and transferred to microtubes containing 1 mL of 5% (*v*/*v*) acetic acid [18]. The experimental procedures for extracting proteins from protein spots and proteolytic cleavage using trypsin to obtain extracts with peptide sequences were performed as described by Vieira et al., 2023 and Santiago et al., 2023 [19,20]. The peptide sequences were characterized by sequence mass spectrometry coupled with liquid chromatography (LC–MS/MS), according to the procedure described by Shevchenko et al., 2006 [29]. Protein identification was performed using Protein Lynx Global Server software (version 2.5) (PLGS) and the UniProt database for Ratus norvegicus species [30].

#### 3.4.2. Expression Analysis by Shotgun–LC–MS/MS

The shotgun-LC–MS/MS strategy was used to obtain the proteome of kidney samples from the C-60 and Hg-60 groups and for protein expression analysis, using the experimental conditions optimized by Shevchenko et al., 2006 and, subsequently, by Bittencourt et al., 2021 [29,30]. The experimental conditions are described as follows: “The protein pellet extracts from kidney tissue samples containing 50 µg of protein were diluted in 50 µL of ammonium bicarbonate 50 mmol L^−1^ and divided into five 10 µL portions, which were mixed with 25 µL Rapigest 0, 2% (*v*/*v*) (Waters Co., Manchester, UK). The mixtures were incubated at 37 °C for 30 min, and then 2.5 µL of dithiothreitol 100 mmol L^−1^ was added and incubated again at 37 °C, for 60 min. After the second incubation step, 2.5 µL of 300 mmol L^−1^ iodoacetamide (Bio-Rad, Hercules, CA, USA) was added for the alkylation of sulfhydryl groups to occur and to maintain the proteins in the primary conformation, and the mixtures were once again incubated for 30 min at room temperature and in the dark. After the third incubation step, protein digestion was performed by adding 10 µL of trypsin (Thermo Fisher, Waltham, MA, USA) for 14 h at 37 °C. The next day, 10 µL of 5% (*v*/*v*) trifluoroacetic acid (Sigma–Aldrich, St. Louis, MO, USA) was added, and the mixtures were incubated for 90 min at 37 °C, ending the proteolytic cleavage step. After the proteolytic cleavage process, the extracts were centrifuged at 14,000 rpm at 6 °C for 30 min. At the end of the process, the supernatants were collected and purified, using C18 spin columns (Thermo Fisher, Waltham, MA, USA). The extracts were then resuspended in 12 µL of antidiuretic hormone (ADH) 1 pmol µL^−1^ + 108 µL of 3% (*v*/*v*) acetonitrile (Sigma–Aldrich, St. Louis, MO, USA) and 3% formic acid (*v*/*v*) (Thermo Fisher, Waltham, MA, USA)”. The peptide extracts were analyzed using a nanoAcquity UPLC Xevo QT of MS system (Waters, Manchester, UK). Protein Lynx Global Server software (version 2.5) (PLGS) was used for peptide analysis and expression analyses. In identifying the peptides, the Monte Carlo algorithm was applied, and a search in the UniProt database for the species Ratus norvegicus was performed [18,30]. For expression analyses (C-60 Group x Hg-60 group), *p* < 0.05 was considered for negatively regulated proteins, and 1—*p* > 0.95 was considered for upregulated proteins. After compiling the results, functional analysis of the differentially expressed proteins was performed using Blas2GO 6.0 software, to determine the functional categories of proteins based on notes from the gene ontology of biological processes [18,31,32,33,34,35].

### 3.5. Statistical Analysis

Statistical analysis was performed, according to the procedures described in previously published papers [9,18,19,20,30,31,32,33,34,35]. However, the statistical analysis procedures are briefly described as follows: “The images of 2D PAGE gels obtained from the renal proteome fractionation experiments (Groups C-60 and Hg-60) were analyzed with ImageMaster 2D Platinum 7.0 software (GE Healthcare). The analyses with ImageMaster 2D Platinum 7.0 software allowed us to obtain correlations between the images of gels (matching), through equivalence analysis between the protein spots in each 2D PAGE run. This also allows a comparison analysis among the protein spots in terms of distribution, volume, relative intensity, isoelectric point and molecular mass. The results of mercury determinations were expressed as M ± SD, and subjected to Student’s *t* test and F test to identify any significant differences, using SAS software (version 8). The significance level was set at 5%, that is, *p* < 0.05”. For protein expression data, statistical analysis was performed using the Protein Lynx Global Server software (version 2.5) (PLGS) of the nanoAcquity UPLC Xevo QTof MS equipment, as described in the previous section.

## 4. Conclusions

The Hg determinations demonstrated that a Hg-bioaccumulation process occurs at a level greater than 700% in the renal proteome of rats exposed to a low concentration of HgCl_2_ for 60 days. The metalloproteomic results indicated that the isoforms of the GST Mu and GPx enzymes and the heat shock proteins, heat shock 70 kDa protein 1-like and heat shock protein beta-1, were expressed only in the group exposed to mercury for 60 days, which qualifies these enzymes/proteins as potential biomarkers of mercurial-species exposure. The actin isoforms were “upregulated” and “downregulated”, and the hemoglobin subunit isoforms were “downregulated”. Additionally, the protein spots in which actin and hemoglobin subunit isoforms were identified showed mercury concentrations in the order of 138 to 750 mg kg^−1^, which qualifies these proteins as possible mercurial-species exposure biomarkers. Notably, the results of the present study corroborate those from the literature reporting the kidney as an organ responsive to intoxication by mercurial species.

## Figures and Tables

**Table 1:** Hg concentration in renal tissue samples and protein pellets from rats exposed to mercury for 30 and 60 days.

Samples	Hg Concentration (µg kg^−1^)
Hg-30 Tissue	39.20 ± 0.71
Hg-60 Tissue	60.40 ± 1.10
Hg-30 Pellet	36.50 ± 0.62
Hg-60 Pellet	55.60 ± 0.98
Fish Liver DOLT 4	2.530 ± 0.060 *

* Hg Concentration—mg kg^−1^; Fish Liver DOLT 4—Certified Concentration = 2.58 ± 0.22 mg kg^−1^. Hg-30—rats exposed to mercury for 30 days; Hg60—rats exposed to mercury for 30 days.

**Table 2:** Proteins identified in mercury-associated protein spots on gels of renal tissue samples from rats exposed to mercury for 60 days (group Hg-60).

Spot ID	Access	Proteins ID	Score	[Hg] (mg kg^−1^)
7		Albumin	58.669	120 ± 2
10	-	Not identified	-	113 ± 2
13	P11517 P02091	Hemoglobin subunit beta-2 Hemoglobin subunit beta-1	272.2158 272.2158	138 ± 2
14	-	Not identified	-	293 ± 4
18	-	Not identified	-	302 ± 5
20	-	Not identified	-	316 ± 5
21	-	Not identified	-	417 ± 6
22	P63259 P60711 P68136 P68035 P63269 P62738	Actin_ cytoplasmic 2 Actin_ cytoplasmic 1 Actin_ alpha skeletal muscle Actin_ alpha cardiac muscle 1 Actin_ gamma-enteric smooth muscle Actin_ aortic smooth muscle	510.8116 510.8116 142.5171 142.5171 142.5171 1425171	750 ± 9
31	P02761	Major urinary protein	212.1593	349 ± 5
35 36	P02091 P11517	Hemoglobin subunit beta-1 Hemoglobin subunit beta-2	333.9544 272.685	84 ± 1

## Data Availability

The data presented in this study are available on request from the corresponding author.

## Supplementary Materials

The following supporting information can be downloaded at: https://www.mdpi.com/article/10.3390/ijms25010164/s1.
