# Supplementary material for: Metalloproteomic Investigation of Hg-Binding Proteins in Renal Tissue of Rats Exposed to Mercury Chloride
Source: Int J Mol Sci. 2023 Dec 21;25(1):164. doi: 10.3390/ijms25010164 (PMC10779082; doi:10.3390/ijms25010164)

Supplementary Materials - International Journal Molecular Sciences

Metalloproteomic investigation of Hg-binding proteins in kidney tissue of rats exposed to mercury chloride

Emerson Carlos de Almeida[1], Victor Diego Faria[1], Felipe Dalmazzo Cirinêu[1], Maria G. A. Santiago[1], Beatriz Miotto[1], José C. S. Vieira[1], Camila Pereira Braga[2], Jiri Adamec[3], Ana A. H. Fernandes[1], Marília A. R. Buzalaf[4], Pedro de Magalhães Padilha[1, 2*]

[1]São Paulo State University (UNESP), Institute of Biosciences, Botucatu-SP, Brazil

[2]University of Nebraska (UNL), Lincoln, United States of America

[3]LSUHSC - School of Medicine, New Orleans, United States of America

[4]University of Sao Paulo, (USP), Bauru, Brazil

Table S1. Proteins identified by shotgun-LS-MS/MS from the pool of renal tissue samples from rats exposed to mercury for 60 days (Hg-60 x C-60 groups). **Unique proteins**.

Accession number	Description	Score PLGS	Unique
P11442	Clathrin heavy chain 1	43.63	CT
P19629	L-lactate dehydrogenase C chain	52.85	CT
Q8CGX0	Insulin-like growth factor 2 mRNA-binding protein 1	53.18	CT
Q63041	Alpha-1-macroglobulin	54.3	CT
Q3SWS9	Janus kinase and microtubule-interacting protein 1	54.33	CT
Q5XI58	Calicin	59.57	CT
P30904	Macrophage migration inhibitory factor	60.28	CT
Q68FT1	Ubiquinone biosynthesis protein COQ9_ mitochondrial	65.66	CT
P27139	Carbonic anhydrase 2	79.33	CT
P07335	Creatine kinase B-type	79.96	CT
P13086	Succinate--CoA ligase [ADP/GDP-forming] subunit alpha_ mitochondrial	82.08	CT
P60705	DNA dC->dU-editing enzyme APOBEC3	91.07	CT
P08010	Glutathione S-transferase Mu 2	94.04	CT
Q5BK56	Glutathione S-transferase Mu 4	94.04	CT
P08009	Glutathione S-transferase Mu 7	94.04	CT
P02680	Fibrinogen gamma chain	104.28	CT
Q5PQL7	Integral membrane protein 2C	109.58	CT
P20761	Ig gamma-2B chain C region	131.31	CT
P62260	14-3-3 protein epsilon	136.57	CT
P35467	Protein S100-A1	137.54	CT
O54912	Potassium channel subfamily K member 3	138.52	CT
P97700	Mitochondrial 2-oxoglutarate/malate carrier protein	179.15	CT
P04041	Glutathione peroxidase 1	209.48	CT

Q6LED0	Histone H3.1	258.18	CT
P84245	Histone H3.3	258.18	CT
P11762	Galectin-1	292.97	CT
P01835	Ig kappa chain C region_ B allele	342.74	CT
P63031	Mitochondrial pyruvate carrier 1	399.01	CT
Q9JJW3	ATP synthase membrane subunit K_ mitochondrial	463.77	CT
Q7TQ16	Cytochrome b-c1 complex subunit 8	752.47	CT
P29418	ATP synthase subunit epsilon_ mitochondrial	773.57	CT
P61016	Cardiac phospholamban	1378.75	CT
P0C6P7	Protein fem-1 homolog B	28.2	Hg60
Q66X93	Staphylococcal nuclease domain-containing protein 1	28.7	Hg60
Q6IG04	Keratin_ type II cytoskeletal 72	33.77	Hg60
Q9ESV1	Leucine zipper protein 1	36.86	Hg60
P15387	Potassium voltage-gated channel subfamily B member 1	37.29	Hg60
P06761	Endoplasmic reticulum chaperone BiP	45.4	Hg60
P70627	Glutamate carboxypeptidase 2	49.65	Hg60
Q63099	Potassium voltage-gated channel subfamily B member 2	52.6	Hg60
Q8CF97	Deubiquitinating protein VCPIP1	56.26	Hg60
P55063	Heat shock 70 kDa protein 1-like	58.37	Hg60
Q4G061	Eukaryotic translation initiation factor 3 subunit B	59.1	Hg60
Q03346	Mitochondrial-processing peptidase subunit beta	70.67	Hg60
P42930	Heat shock protein beta-1	82.52	Hg60
Q9QZC4	Choline-phosphate cytidylyltransferase B	90.76	Hg60
P11661	NADH-ubiquinone oxidoreductase chain 5	93.42	Hg60
P29266	3-hydroxyisobutyrate dehydrogenase_ mitochondrial	96.81	Hg60
P07943	Aldo-keto reductase family 1 member B1	106.68	Hg60
P09117	Fructose-bisphosphate aldolase C	185.48	Hg60

P70623	Fatty acid-binding protein_ adipocyte	358.89	Hg60
Q5M9I5	Cytochrome b-c1 complex subunit 6_ mitochondrial	614.64	Hg60

Proteins that were expressed in only one treatment.

Table S2. Proteins identified by shotgun-LS-MS/MS from the pool of renal tissue samples from rats exposed to mercury for 60 days (Hg-60 x C-60 groups). **Upregulated.**

Accession number	Description	Score PLGS	Hg60 x CT_Ratio	Hg60 x CT__P
P48500	Triosephosphate isomerase	883.08	1.094174288	0.96
P20759	Ig gamma-1 chain C region	30.14	1.786038401	0.97
P17764	Acetyl-CoA acetyltransferase_ mitochondrial	400.61	1.083287066	0.98
P02600	Myosin light chain 1/3_ skeletal muscle isoform	831.88	1.246076729	0.98
Q01205	Dihydrolipoyllysine-residue succinyltransferase component of 2-oxoglutarate dehydrogenase complex_ mitochondrial	84.42	1.271249144	0.99
P51868	Calsequestrin-2	258.94	1.221402762	0.99
P56571	ES1 protein homolog_ mitochondrial	245.35	1.363425117	1
P10817	Cytochrome c oxidase subunit 6A2_ mitochondrial (Fragment)	418.33	1.390968147	1
Q64119	Myosin light polypeptide 6	945.5	1.258600015	1
P23928	Alpha-crystallin B chain	2935.82	1.094174288	1
P56574	Isocitrate dehydrogenase [NADP]_ mitochondrial	3019.73	1.138828378	1
P02564	Myosin-7	9904.11	1.127496849	1
P68035	Actin_ alpha cardiac muscle 1	15574.29	1.173510867	1

Up *= Proteins with a 'p' value greater than 0.95*

Table S3. Proteins identified by shotgun-LS-MS/MS from the pool of renal tissue samples from rats exposed to mercury for 60 days (Hg-60 x C-60 groups). **Downregulated.**

Accession number	Description	Score PLGS	Hg60 x CT_Ratio	Hg60 x CT__P
P09811	Glycogen phosphorylase_ liver form	53.91	0.778800783	0
P48721	Stress-70 protein_ mitochondrial	71.86	0.748263574	0
P85834	Elongation factor Tu_ mitochondrial	91.21	0.612626388	0
Q64578	Sarcoplasmic/endoplasmic reticulum calcium ATPase 1	139.31	0.835270205	0
P17209	Myosin light chain 4	234.32	0.763379486	0
P09812	Glycogen phosphorylase_ muscle form	249.52	0.886920439	0
P11507	Sarcoplasmic/endoplasmic reticulum calcium ATPase 2	272.57	0.852143792	0
Q68FU3	Electron transfer flavoprotein subunit beta	360.5	0.869358235	0
P04764	Alpha-enolase	503.99	0.835270205	0
Q68FY0	Cytochrome b-c1 complex subunit 1_ mitochondrial	686.57	0.852143792	0
P04642	L-lactate dehydrogenase A chain	1034.95	0.869358235	0
P16036	Phosphate carrier protein_ mitochondrial	1048.55	0.771051593	0
P10888	Cytochrome c oxidase subunit 4 isoform 1_ mitochondrial	1068.03	0.748263574	0
P62804	Histone H4	1544.51	0.852143792	0
D3ZAF6	ATP synthase subunit f_ mitochondrial	1651.57	0.843664815	0
Q06647	ATP synthase subunit O_ mitochondrial	2620.75	0.886920439	0
P23693	Troponin I_ cardiac muscle	4084.66	0.826959136	0
Q29RW1	Myosin-4	5297.91	0.826959136	0
P60711	Actin_ cytoplasmic 1	8304.34	0.740818212	0
P63259	Actin_ cytoplasmic 2	8330.96	0.740818212	0
P63269	Actin_ gamma-enteric smooth muscle	12264.11	0.740818212	0
P62738	Actin_ aortic smooth muscle	13364.2	0.755783741	0
Q9QZ76	Myoglobin	13819.06	0.923116348	0

P68136	Actin_ alpha skeletal muscle	14669.85	0.755783741	0
P11517	Hemoglobin subunit beta-2	17600.93	0.740818212	0
P16409	Myosin light chain 3	22870.06	0.904837417	0
P02091	Hemoglobin subunit beta-1	25672.54	0.786627865	0
P01946	Hemoglobin subunit alpha-1/2	31159.12	0.71177032	0
P53534	Glycogen phosphorylase_ brain form (Fragment)	125.57	0.810584251	0.01
P16617	Phosphoglycerate kinase 1	157.06	0.852143792	0.01
P07632	Superoxide dismutase [Cu-Zn]	159.66	0.637628159	0.01
P00507	Aspartate aminotransferase_ mitochondrial	1740.83	0.913931182	0.01
P15999	ATP synthase subunit alpha_ mitochondrial	8218.37	0.951229424	0.01
P10719	ATP synthase subunit beta_ mitochondrial	11155.46	0.941764535	0.01
P63018	Heat shock cognate 71 kDa protein	133.72	0.878095435	0.02
B0BNN3	Carbonic anhydrase 1	238.38	0.58274824	0.03
P12346	Serotransferrin	353.39	0.886920439	0.03
P48675	Desmin	764.48	0.923116348	0.03
O88989	Malate dehydrogenase_ cytoplasmic	1362.02	0.923116348	0.03
P19511	ATP synthase F(0) complex subunit B1_ mitochondrial	1545.46	0.895834136	0.03
P42123	L-lactate dehydrogenase B chain	4618.54	0.93239382	0.03
P18596	Sarcoplasmic/endoplasmic reticulum calcium ATPase 3	25.58	0.860707971	0.04
P0CG51	Polyubiquitin-B	126.25	0.733446954	0.04
P62986	Ubiquitin-60S ribosomal protein L40	140.04	0.771051593	0.04
P31399	ATP synthase subunit d_ mitochondrial	591.86	0.895834136	0.04
Q6PDU7	ATP synthase subunit g_ mitochondrial	1347.26	0.886920439	0.04
Q9ER34	Aconitate hydratase_ mitochondrial	2861.81	0.96078944	0.04

Down *= Proteins with a 'p' value less than 0.05.*

Original Images of the 2D PAGE Gels

Kidney – Control Group 60 days/C-60

Gel A

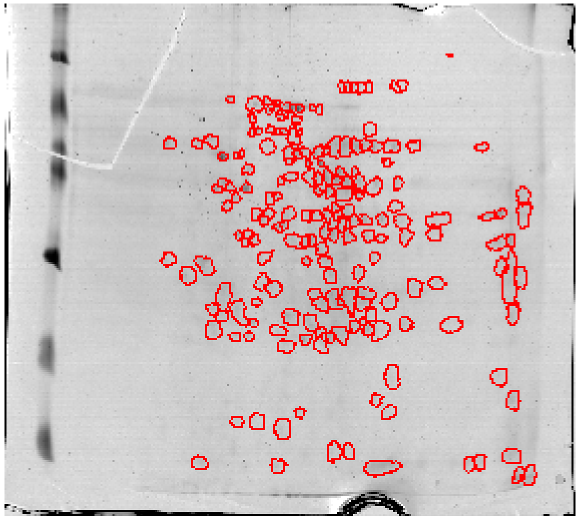

Gel b

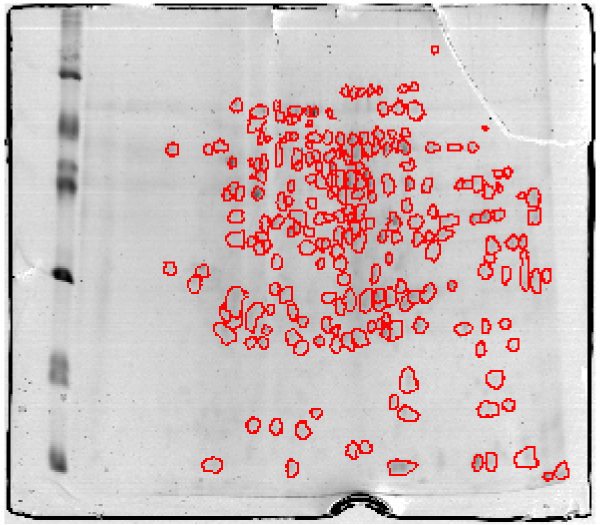

Gel C

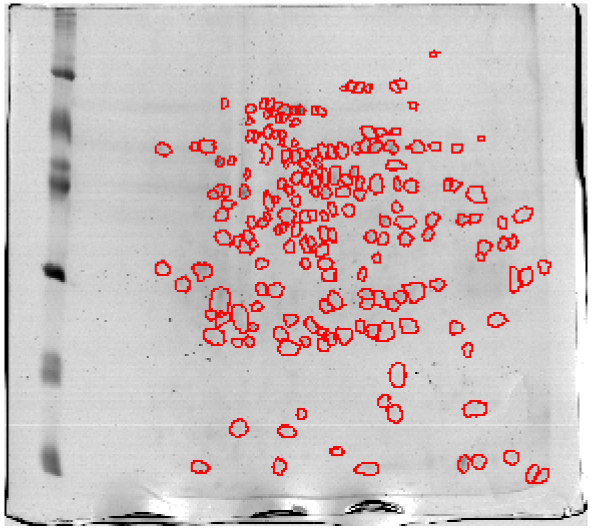

Kidney – Mercury Group 60 days/Hg-60

Gel A

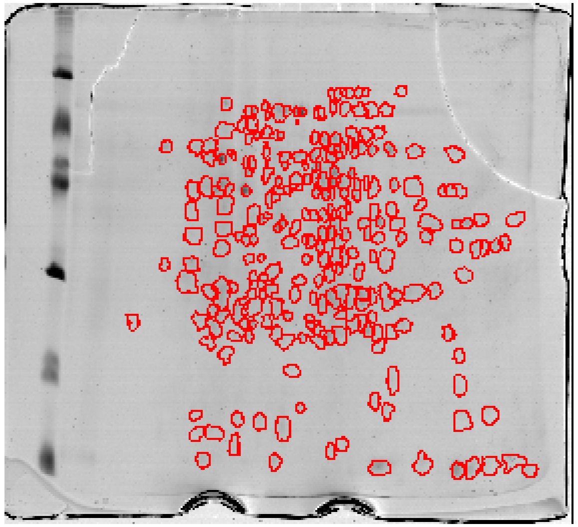

Gel B

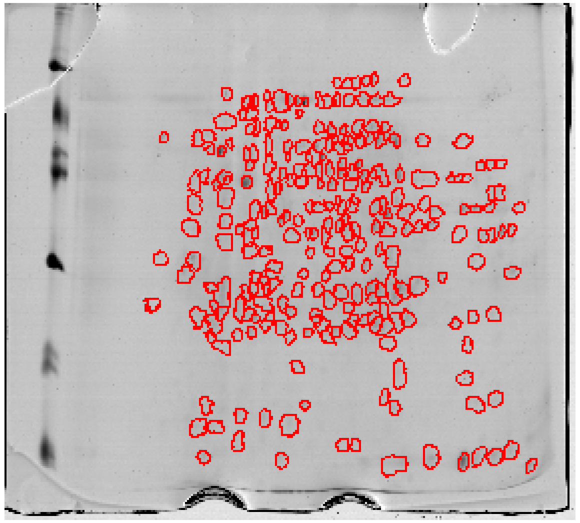

Gel C

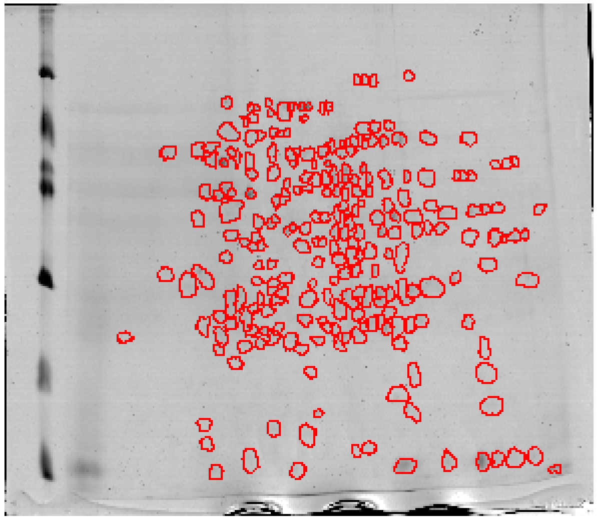

Supplement: Supplementary file 1 [file ijms-25-00164-s001.zip › ijms-2709678-supplementary.pdf]
